# Are explicit and implicit affective attitudes toward different body shape categories related to the own body-satisfaction in young women? The role of mindfulness, self-compassion and social media activity

**DOI:** 10.1007/s00426-021-01536-z

**Published:** 2021-06-11

**Authors:** Petra Jansen, Franziska Anna Schroter, Philipp Hofmann

**Affiliations:** Faculty of Human Sciences, University of Regensburg, Universitätstraße 31, 93053 Regensburg, Germany

## Abstract

Implicit and explicit attitudes influence our behavior. Accordingly, it was the main goal of the paper to investigate if those attitudes are related to body image satisfaction. 134 young women between 18 and 34 years completed an explicit affective rating and an implicit affective priming task with pictures of women with different BMIs. Because it is well known that mindfulness, self-compassion and social media activity influence body image satisfaction, these variables were registered as well. The results confirmed an explicit positive affective bias toward pictures of slim women and a negative bias toward emaciated and obese body pictures. It adds to the literature that the explicit positive bias does not hold true for the strongest form of underweight, suggesting that instead of dividing different body shapes into two groups, different gradings of under- and overweight should be considered. Concerning the affective priming task, no significant differences between the different pictures could be carved out. Implicit and explicit affective attitudes were not related to the body satisfaction of the participating women. In line with former studies, body satisfaction was predicted by the actual-ideal weight discrepancy, the BMI, aspects of mindfulness and self-compassion. This study indicates that implicit and explicit affective attitudes toward underweight and overweight women are unrelated to the participants’ body satisfaction.

## Introduction

Body image describes “a person’s perceptions, thoughts and feelings about his or her body” (Grogan, 2008). According to Heider et al. (2018), body image dissatisfaction results from a discrepancy between the perceived actual body image and the ideal body image, which can be described as internalized ideals about the own physical appearance. Especially women suffer from body dissatisfaction, e.g., it had a significantly higher impact on their self-esteem compared to men in a study with 235 participants (Furnham et al., 2002; Karazsia et al., 2017). Furthermore, a body dissatisfaction-eating disorder symptomatology relationship exists with the moderating variables neuroticism and body surveillance (Brannan & Petrie, 2008). Regarding the positive connection between body satisfaction and quality of life, it is important to investigate factors which are related to body satisfaction. Here the implicit and explicit affective attitudes toward under- and overweight and the components of mindfulness, self-compassion and the social media use will be investigated in detail.

### Attitudes toward different under- and overweight categories

One interesting question is, how the own general attitudes toward under- and overweight are related to body satisfaction. It has been discussed whether an “anti-fat” bias exist in society, if it is more pronounced in women or men and if it is related to other concepts like for example intelligence (Nolan et al., 2013). It has been demonstrated that the bias toward weight concepts was higher in the implicit tasks (measured with the Implicit Association Test (IAT) and the Implicit Relational Assessment Procedure (IRAP) compared to the explicit measures and that the bias was rather a pro-slim than an anti-fat effect (Roddy et al., 2009). However in another study the authors showed a strong explicit pro-slim and anti-fat bias and no implicit weight bias in female Spanish college students (Maroto Expósito et al., 2015). The differences in these studies might be attributable to the different cultural contexts, e.g., Spanish women score substantially lower on body dissatisfaction compared to Euro-American women (Warren et al., 2008). The relation of implicit attitudes toward thinness and fatness with body satisfaction has—to our best knowledge—only been investigated in the following three studies: first, it has been demonstrated that the implicit desire to be thin tended to be more pronounced in participants, who were implicitly more dissatisfied with their body (Heider et al., 2018), which is in line with the results of a former study (Heider et al., 2015). In their study conducted in Belgium, the IRAP has been used to investigate implicit beliefs. Second, it has been shown that Spanish women with low and high body dissatisfaction had equally positive implicit attitudes toward thinness (Hernández-López et al., 2019). However, only the group of women with low body dissatisfaction showed implicit positive attitudes toward fatness. The authors concluded that the focus of prevention for eating disorders should not be on changing the attitudes toward thinness but on promoting the appreciation of larger body shapes. In the third study carried out in Canada, it has been demonstrated that body-dissatisfied women attended to both fat and thin-related words more than body-satisfied women (Tobin et al., 2018). The exposure to thin models did not increase this effect. In those three studies, the implicit attitudes toward fatness and thinness were measured with more cognitive implicit measurements like the IAT or the IRAP, but the results differ due to the research question investigated. In general, for body dissatisfied women, the processing of any weight-related information might be affected by negative weight-related schemas (Tobin et al., 2018), while experiencing a higher drive for thinness (Heider et al., 2018). However, both, women with a lower and higher body dissatisfaction had positive implicit attitudes toward thinness (Hernández-López et al., 2019).

As mentioned above, all three studies used cognitive implicit measurements. Because it is well known that body image is related to emotional problems (Ren et al., 2018) and trait emotional intelligence is related to the actual-ideal weight discepancy and body appreciation (Swami et al., 2010), it is worth investigating not only the cognitive aspects of implicit and explicit measurements but also the affective ones. This is important, because affective implicit attitudes have been found to be a central aspect for decision-making, for example it has been demonstrated that the affective implicit attitudes are the main driver for food choices when cognitive resources are limited. The cognitive component of implicit attitudes influences food choices for participants low in impulsivity when the cognitive resources are high (Trendel & Werle, 2016).

### Implicit and explicit affective attitudes

As mentioned above and in contrast to the IAT, which mainly reflects associations between concepts and related cognitive representations of feelings, the affective priming task has an additional somato-affective component (Brand & Ekkekakis, 2018; deHouwer et al., 2009). Affective priming effects reflect the participants’ implicit attitudes toward a primed object or person (deHouwer et al., 2009). Various explanations for the affective priming effect have been discussed in the past. The “spreading activation mechanism” approach proposes that if prime and target have a congruent valence, response initiation will be facilitated because the respective pathway has already been activated (deHouwer et al., 2009). In case of a non-congruent prime-target pair, the wrong response pathway will be activated, so it needs to be inhibited first before the correct response can be triggered (Fazio, 2001). Other possible explanations include the assumption of a connectivity network (Spruyt et al., 2002) or the theory of target pre-activation through semantic or response priming (Eder et al., 2012). To investigate the affective attitudes toward persons or objects, it seemed to be relevant to use this implicit measurement procedure. In a further study, it has been demonstrated that a specific form of mindfulness, a brief loving kindness meditation, increases social connection toward strangers (Hutcherson et al., 2008). Their study showed that even after only a few minutes of loving-kindness meditation, the feelings of social connection and positivity toward novel individuals on both implicit and explicit levels were ameliorated. To our best knowledge, the implicit affective priming paradigm has not been used in relation to the concepts of thinness and fatness.

Body dissatisfaction is an important topic for young women, especially regarding its connection to eating disorders which were found to experience a drastic increase in prevalence throughout the last years (Brannan & Petrie, 2008; Galmiche et al., 2019). Consequently, it is worth asking which relevant factors can improve positive attitudes toward the own body, and which factors have a deteriorating effect. There are a lot of bio-psycho-social risk factors of body dissatisfaction, we have chosen three of them which have gained a considerable amount of attention in relation to body image research in the last years: on one side aspects of mindfulness and self-compassion and on the other side social media use.

### Factors influencing body satisfaction

Next to the own BMI which correlated negatively with the body image in a study with young men (Watkins et al., 2008) there are other factors, which influence the body image: first, a positive relation was shown between dispositional mindfulness and body satisfaction. Mindfulness is described as the ability to be in the present moment without judging the situation (Kabat-Zinn, 1990). Previous research suggests that attention regulation, body awareness, emotion regulation and change in perspective are the mechanisms behind mindfulness (Hölzel et al., 2011). Mindfulness interventions include various kinds of practices, such as attentional, constructive and deconstructive meditations (Dahl et al., 2015). Individuals who were found to be more mindful engaged less often in body comparison. Also, there was a relation between mindfulness and body satisfaction which was mediated by body comparison (Dijkstra & Barelds, 2011). It has been demonstrated that mindfulness skills like describing, accepting without judgment and acting with awareness were associated with a higher body satisfaction, while higher scores in the mindfulness aspect of observing were connected to higher eating disorder symptoms in a non-clinical sample (Prowse et al., 2013). The importance of mindfulness has also been shown in an experimental design: it was found that an eight-week mindfulness-based cognitive therapy program reduced, amongst others, concerns about body image in women with problematic eating behavior (Alberts et al., 2012). In another study, 81 students who suffered from an eating disorder were randomly assigned to either a brief mindful breathing exercise or a resting control condition (Keng & Ang, 2019). Subsequently, they had to complete an explicit body dissatisfaction questionnaire, the IAT with body-related words, and a negative affect scale. While the brief mindfulness induction led to a reduced negative affect and to a trend for a lower explicit body dissatisfaction, there was no such effect for the implicit body image, which gives a hint that mindfulness interventions influence explicit and implicit attitudes in a different manner.

Second, it was shown that self-compassion was positively related to body satisfaction (Wasylkiw et al., 2012). Self-compassion describes the ability to see oneself as a good friend (Neff & Dahm, 2015). Furthermore, self-compassion has been linked to lower media and interpersonal thinness pressure, thin-ideal internalization, social appearance comparisons, body surveillance, body shame, body satisfaction, and drive for thinness (Braun et al., 2016). Next to this, the influence of an one-week self-compassion meditation training for body image satisfaction in young adult women was investigated in the past (Toole & Craighead, 2016). Among others, the authors investigated body shame, body surveillance, body appreciation and body satisfaction. They demonstrated that only in the dimensions body appreciation and body surveillance an improvement from pre- to posttest could be shown for the intervention group.

Furthermore, self-compassion seems to offer a novel way for working against the negative impact of social media on the body satisfaction of women (Slater et al., 2017). This is important, because the maladaptive effect of the use of social networking websites on body image has been demonstrated in a systematic review including 20 studies (Holland & Tiggemann, 2016). These measures included the overall time spent on social media, the frequency and the number of social media friends/followers. Gender did not moderate these results. Uploading photos and provoking negative feedback via status updates has been identified as problematic. Also, viewing the photos of other persons were correlated with body weight dissatisfaction and drive for thinness. In addition, the effect of social grooming behaviors (“liking”) was related to the drive to become thinner.

### Main goal of this study

The main goal of the paper is to investigate the explicit and implicit affective attitudes toward under-, normal- and overweight women in a sample of young women. Second, the relation to body satisfaction and the contributing factors will be investigated. The following hypotheses can be formulated:

1. We assume an implicit (Roddy et al., 2009) and explicit (Maroto Expósito et al., 2015) bias toward pro-slim instead of anti-fat body shapes in women, measured with an explicit rating task and an implicit affective priming paradigm, in that form that the pictures of underweight women are rated implicitly and explicitly as more positive than the ones of overweight women. Our study will extend the results of Maroto Expósito et al. (2015) because, in this study it was not differentiated between different forms of under- and overweight. Furthermore, if at all, only a small correlation is expected between implicit and explicit measurements (Cameron et al., 2012).
2. In line with the study of Hernández-López et al. (2019), we assume a relation between implicit attitudes toward thinness and fatness and body satisfaction.
3. We hypothesize that self-compassion (Braun et al., 2016), the mindfulness aspects of acting with awareness, describing and accepting without judgment (Prowse et al., 2013), and the attitudes toward women with different BMIs are positively associated with body satisfaction, whereas the use of social media shows a negative relation (Holland & Tiggemann, 2016). According to the study of Watkins et al., (2008), we will include the own BMI as well as the actual-ideal weight discrepancy (Swami et al., 2008) as two other relevant variables, which are expected to negatively predict body satisfaction.

## Methods

### Participants

Based on the results of Roddy et al. (2009), a medium effect size for the difference between the ratings of the different female figures was expected. Accordingly, with a medium effect size *f* = 0.25, an alpha-level of *p* = 0.05 and a power of 1 − *β* = 0.95, a power analysis with G*power (Faul et al., 2007) for the repeated measures ANOVA resulted in *N* = 31 to detect significant effects in the explicit, as well as the implicit attitudes between the pictures of the different BMI groups. Regarding our third hypothesis, the most relevant predictors (based on correlational analyses) will be entered into a regression analysis for the dependent variable body satisfaction. With a medium effect size *f* = 0.15, an alpha-level of *p* = 0.05, a power of 1 − *β* = 0.80 and 18 predictors (positive and negative scale of self-compassion, 5 different explicit and implicit attitudes toward the emaciated, underweight, normal weight, overweight and obese pictures, the use of social media (here: time spent per day on social media), own BMI, actual-ideal weight discrepancy, and the three mindfulness aspects of acting with awareness, accepting without judgment and describing), a power analysis for the linear regression resulted in a sample size of *N* = 150 women.

152 participants completed the study. We had to exclude eight participants because they only had “none” responses in the explicit or the implicit tasks. Reasons for “none” responses could be either not answering the items or using the wrong keys instead of the arrow keys. Seven participants were excluded because they had more than 50% errors in the implicit task. Another two participants had to be excluded because they indicated to be male. Because body appreciation changes with age (Tiggemann & McCourt, 2013) one participant was excluded because she was over 35 years old. The remaining 134 participants were exclusively female and between 18 and 34 years old (*M* = 22.81, *SD* = 3.33). Only two students had a mother tongue other than German. None of the participants were emaciated (BMI < 15), 8 women had an underweight BMI (< 18.5), 113 women had a normal weight BMI (< 25), 10 women had an overweight BMI (< 30), and 2 women an obese BMI (≥ 30). One woman did not indicate her weight and height. Participants were recruited via the newsletter of the University and social media. Students received course credit for their participation. 84.3% were college students, 2.2% high-school students, 8.2% had a full-time job, 4.5% a part time job and one woman stayed at home.

All procedures were in accordance with the ethical standards of the institutional and/or national research committee and with the 1964 Helsinki declaration and its later amendments or comparable ethical standards. Informed consent was obtained from all individual participants included in the study.

### Material

#### Demographic questionnaire

Our demographic questionnaire included items concerning mother tongue, age, meditation experience (years, hours per week), yoga experience (years, hours per week) sport participation in hours per week, social media (facebook, twitter, instagram) use (hours per day, mostly used social media platform, number of social media friends/followers), self-reported weight and height (the body mass index, BMI, was calculated by kg/m^2^), see Table 1. For our further inferential statistics, missing values of relevant variables (BMI = 0.75% missing; time spent on social media = 4.48% missing) were imputed with the mean of the sample.

**Table 1:** Demographic data of the participating women (means and SD)

Age (*M*, SD)	Meditation Experience (%)	Yoga Experience (%)	Sports activity in h/week (*M*, SD)	Social media (*M*, SD)	Own BMI (*M*, SD, range)
22.81 (3.33)	14.9% never, 45.5% once, 20.9% a few times a year 7.5% a few times a month 6.7% a few times a week 4.5% everyday	6.0% never, 26.1% once, 21.6% a few times a year 25.4% a few times a month 14.9% a few times a week 5.2% everyday	6.23 (3.55)	Time spent in h: 1.93 (1.09) Followers/friends: 419.95 (347.20)	21.76 (2.51, 17.94–35.17)

#### Multidimensional Body-Self Relations Questionnaire—Appearance Scales (MBSRQ; Cash, 2018; German Version: Vossbeck-Elsebusch et al., 2014)

The appearance scales of the Multidimensional Body-Self Relations Questionnaire (MBSRQ-AS; Cash, 2018) consist of 34 items which assess only appearance-related aspects of body image. The questionnaire includes the following 5 scales: appearance evaluation (7 items, example: “My body is sexually appealing”), appearance orientation (12 items, example: “Before going out in public, I always notice how I look”), body areas satisfaction (9 items, using a 1 to 5 scale to indicate how dissatisfied or satisfied you are with each of the following areas or aspects of your body), overweight preoccupation (4 items, example: “I constantly worry about being or becoming fat”) and self-classified weight (2 items, example: “ I think I am (Very Underweight/Somewhat Underweight/Normal Weight/Somewhat Overweight/Very Overweight)”. Items are rated on a 5-point Likert scale. Most items measure agreement (1 = “Definitely disagree” to 5 = “Definitely agree”), satisfaction (1 = “Very dissatisfied” to 5 = “Very satisfied”) or frequency (1 = “Never” to 5 = “Very often”). The Self-Classified Weight scale has five specific response options (1 = “Very underweight” to 5 = “Very overweight”). The cognitive aspects of body satisfaction were assessed with the body area satisfaction scale of the MBSRQ in past studies (Arbour & Ginis, 2006). Furthermore, the body appearance evaluation scale was chosen as a second criterion to measure body satisfaction because it measures the feeling of physical attractiveness but does not tap satisfaction with discrete aspects of the own appearance. Cronbach’s alpha ranged from *α* = 0.74 to *α* = 0.91 in a female sample (Cash, 2000). In the study here, Cronbach’s alpha varied between *α* = 0.66 to *α* = 0.82. Only the scale “self-classified weight” with two items has a Cronbach’s alpha below *α* = 0.7.

#### Self-compassion scale (SCS, Neff, 2003; German version, Hupfeld & Ruffieux, 2011)

The SCS measures how someone acts toward him-/herself in difficult times and it consists of three positive (self-kindness, example: “I try to be loving to myself when I am feeling emotional pain”, common humanity, example: “When things are going badly for me, I see the difficulties as part of life that everyone goes through”, mindfulness, example: “When something upsets me, I try to keep my emotions in balance”) and three negative (self-judgement, example: “When time are really difficult I tend to be tough on myself”, isolation, example: “When I’m feeling down, I tend to feel like most other people are propable happier than I am”, over-identification, example: “When something upsets me I get carried away with my feelings”) subscales, which can be built by computing the mean over the respective items. Each item is rated on a 5-point Likert scale, ranging from 1 = “Almost never” to 5 = “Almost always”. The overall score reached an internal validity of *α* = 0.92 for the original English scale, the Cronbach’s alpha of the six subscales varied between 0.75 and 0.81 (Neff, 2003). The German version was validated in two samples with 396 and 165 participants, revealing a Cronbach’s Alpha of *α* = 0.66–*α* = 0.83 for the subscales and 0.91 for the overall score (Hupfeld & Ruffieux, 2011). However, the one-factor structure of the total score seemed not to be warranted in a German scale, but the positive and negative scale showed a reasonable fit (Coroiu et al., 2018). Cronbach’s alpha in the present study for the positive scale was *α* = 0.89 and for the negative scale *α* = 0.91.

#### Five-Facet Mindfulness Questionnaire (FFMQ; Baer et al., 2008; German-version: Michalak et al., 2016)

The FFMQ is composed of five sub-dimensions, namely observing (example: “When I take a shower or bath, I stay alert to the sensations of water on my body”), non-reactivity (example: “I watch my feeling without getting lost in them”), acting with awareness (example: “I find myself doing things without paying attention”), non-judging (example: “I disapprove of myself when I have irrational ideas”) and describing (example: “I can usually describe how I feel at the moment in considerable detail”). Every subscale includes seven to eight items, summing up to an overall item number of 39. Participants rate the items on a 5-point Likert scale, ranging from 1 = “Applies very rarely” to 5 = “Applies very often”. A study with 550 German college students found internal consistencies between 0.74 and 0.90 for the subdimensions (Michalak et al., 2016). In the study presented here Cronbach’s alpha of the five sub-scales varied between *α* = 0.66 and *α* = 0.91. Only the dimension “observe” showed a reliability below *α* = 0.70.

#### Photographic Figure Rating Scale (PFRS; Swami et al., 2008; Austrian-version: Swami et al., 2012)

**Fig. 1:**
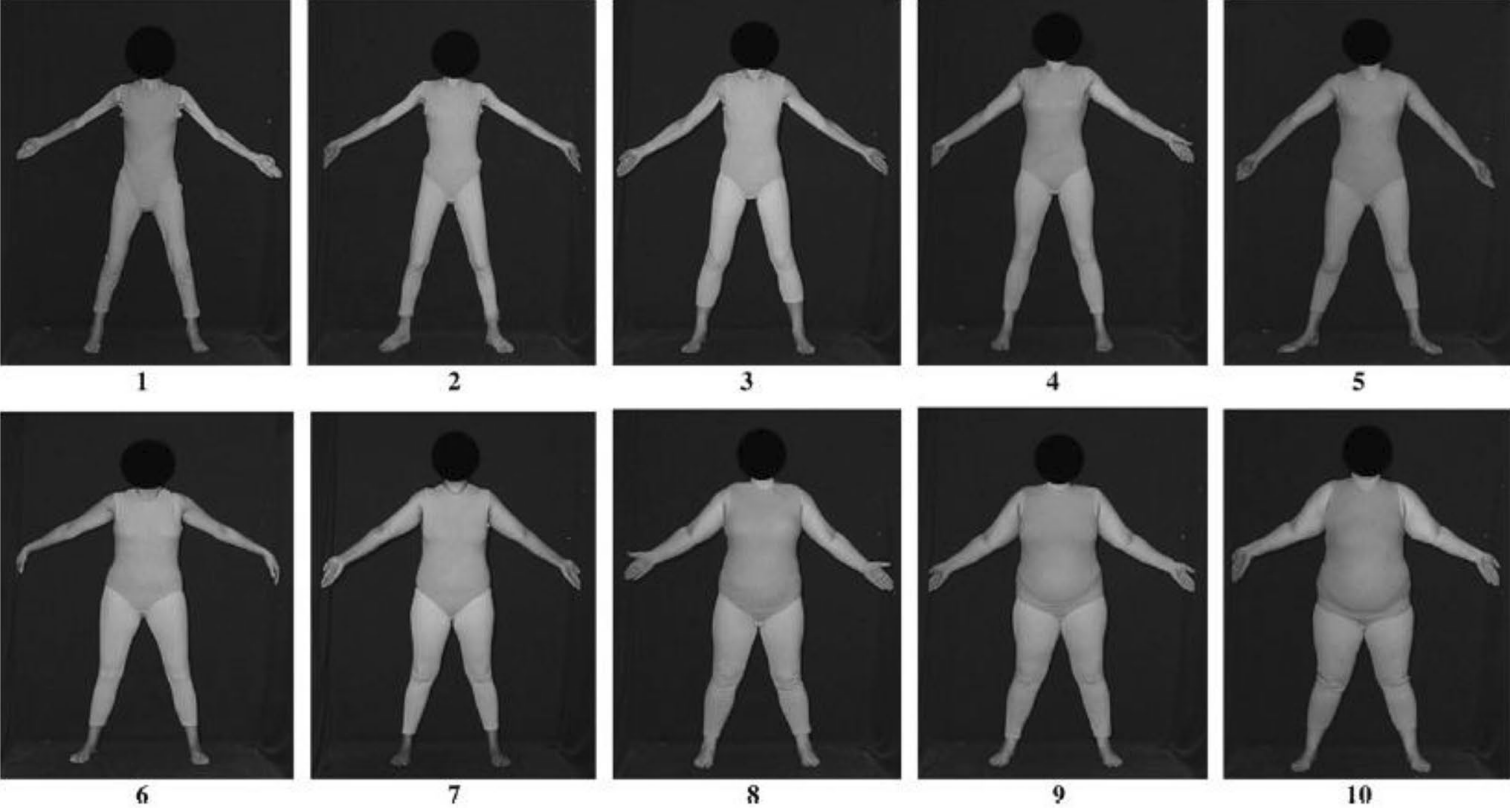
Image used in the PFR-scale, rights obtained from the paper of Swami et al. (2008)

Body image was further investigated with the Photographic Figure Rating Scale (PFRS). This scale consists of ten photographic images of real women varying in BMI (Swami et al., 2008, 2012). The pictures show ten women, with the same clothing and body posture and without showing the face. Five different BMI categories are represented by two pictures each: emaciated BMI (< 15), underweight BMI (< 18.5), normal weight BMI (< 25), overweight BMI (< 30), and obese BMI (≥ 30), see Fig. 1. Participants had to choose the figure, (a) which they found most attractive, which is the (b) biggest or (c) thinnest body shape they would still rate as attractive, (d) they thought men would rate as most attractive, (e) resembles their own body shape most and (f) they considered to be their ideal body shape. The actual-ideal weight discrepancy was measured by subtracting ideal ratings (f) from current self-ratings (e). For this test, a good construct validity was obtained as well as an adequate test–retest reliability (Swami et al., 2008). Cronbach’s alpha in the present study was *α* = 0.78.

#### Explicit affective attitudes

For the explicit rating task, ten pictures from the PFRS were used, see description above (Swami et al., 2008, 2012). To each picture, three questions were asked in the explicit rating task concerning the attitude, the similarity, and the closeness the participant felt toward the women in the picture (Hutcherson et al., 2008). The questions were asked in a random order and participants had 5 seconds to answer, to ensure a spontaneous response. Each item was rated on a 7-point Likert scale. According to Hutcherson et al. (2008), a composite score was calculated by the mean of the answers to the three questions.

#### Implicit affective attitudes

**Fig. 2:**
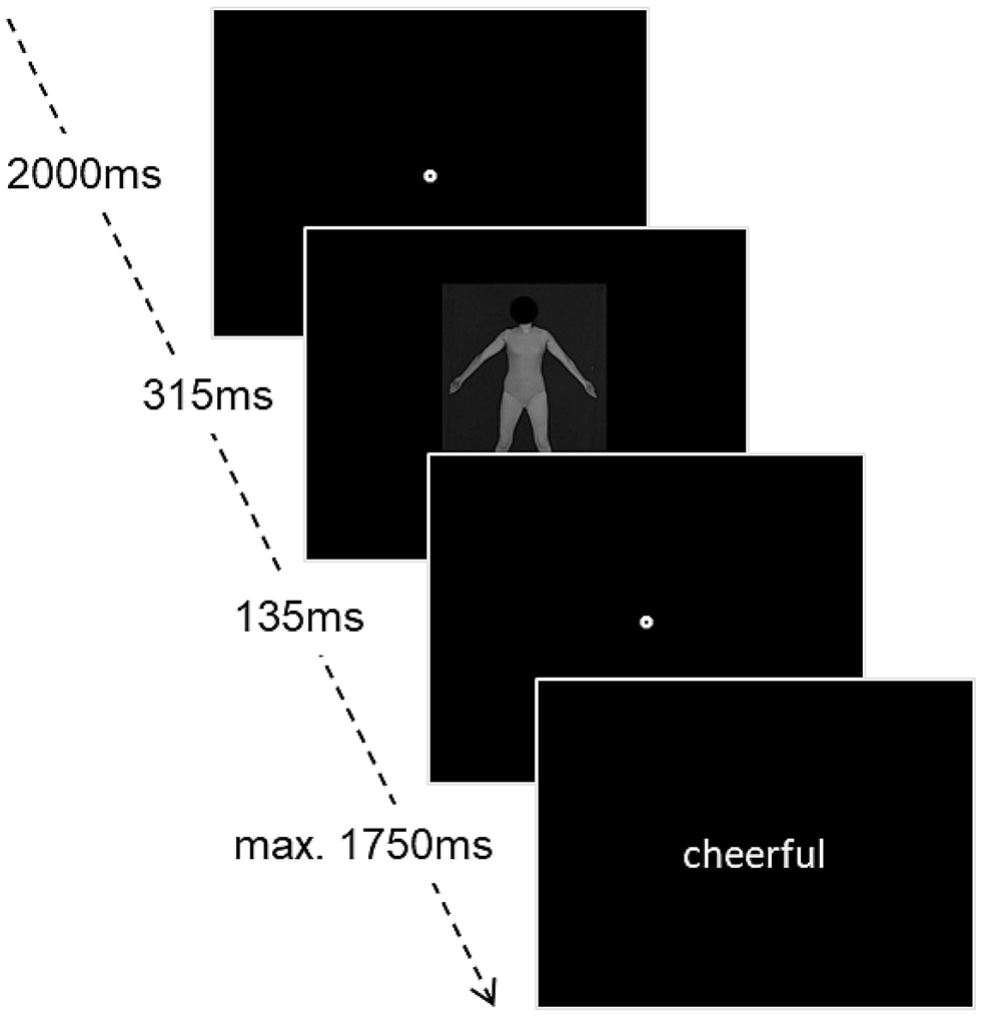
Experimental setup

To assess the implicit attitudes, an affective priming task was implemented (Fazio et al., 1995; Hutcherson et al., 2008) using the same ten pictures from the PFRS. Before the main block, a short practice trial was added with four pictures of neutral looking strangers. Each trial started with a fixation point in the middle of the screen, which was presented for 2000 ms. Subsequently, a picture of a woman was shown for the duration of 315 ms, followed by another fixation point for 135 ms. Finally a word was presented on the screen, randomly picked from a set of ten negative and ten positive words, which were retrieved from the Berlin Affective Word List (BAWL-R) (Võ et al., 2009). Participants were instructed to indicate if the word was positive or negative, using the arrow keys. The word was shown for a maximum duration of 1750 ms (Fig. 2). In case the participant failed to respond in time, the trial was repeated later.

On average, participants made 1.33 (SD = 1.89) mistakes. Error trials (6.6%) were imputed with the mean of the correct trials. As an indicator for the implicit attitude toward the different female body shapes, the mean reaction time for positive words was subtracted from the mean reaction time for negative words, so a higher difference score reflected a more positive implicit evaluation (Hutcherson et al., 2008).

### Procedure

The study was implemented as an online experiment on the platform Jatos.org. It was created using the programs Open Sesame and SurveyJS. The whole experiment lasted about 20 min. In the beginning, demographical data, body satisfaction, self-compassion, and mindfulness were surveyed. Subsequently, the PFRS, the explicit and the implicit tasks were conducted, all tasks following the order in which they were mentioned in this section. Participants had to use their computer or laptop to ensure that the experiment is running correctly.

### Statistical analysis

To test if there is a difference between the explicit ratings of the pictures of the women with different BMIs, a repeated measures ANOVA was conducted with the within subject factor “figure” (emaciated, underweight, normal weight, overweight and obese women). Subsequently, it was analyzed if there is a difference in the implicit affective ratings of the pictures of the women with different BMIs, using a repeated measures ANOVA which was conducted for the difference scores (reaction time) between negative and positive words with the within factor “figure”. If sphericity was violated, data was Greenhouse-Geiser corrected if ε was beyond 0.75 and Huynh–Feldt corrected if ε was above 0.75. Furthermore, a Pearson correlation between the explicit and implicit ratings was conducted. After this, stepwise linear regressions with the 18 predictors mentioned above were calculated for the body area satisfaction and the body appearance evaluation scale. Only the significant predictors were presented due to readability. The reasons why these two scales were used as criterions are indicated in the method section.

## Results

### Explicit and implicit affective attitudes

**Fig. 3:**
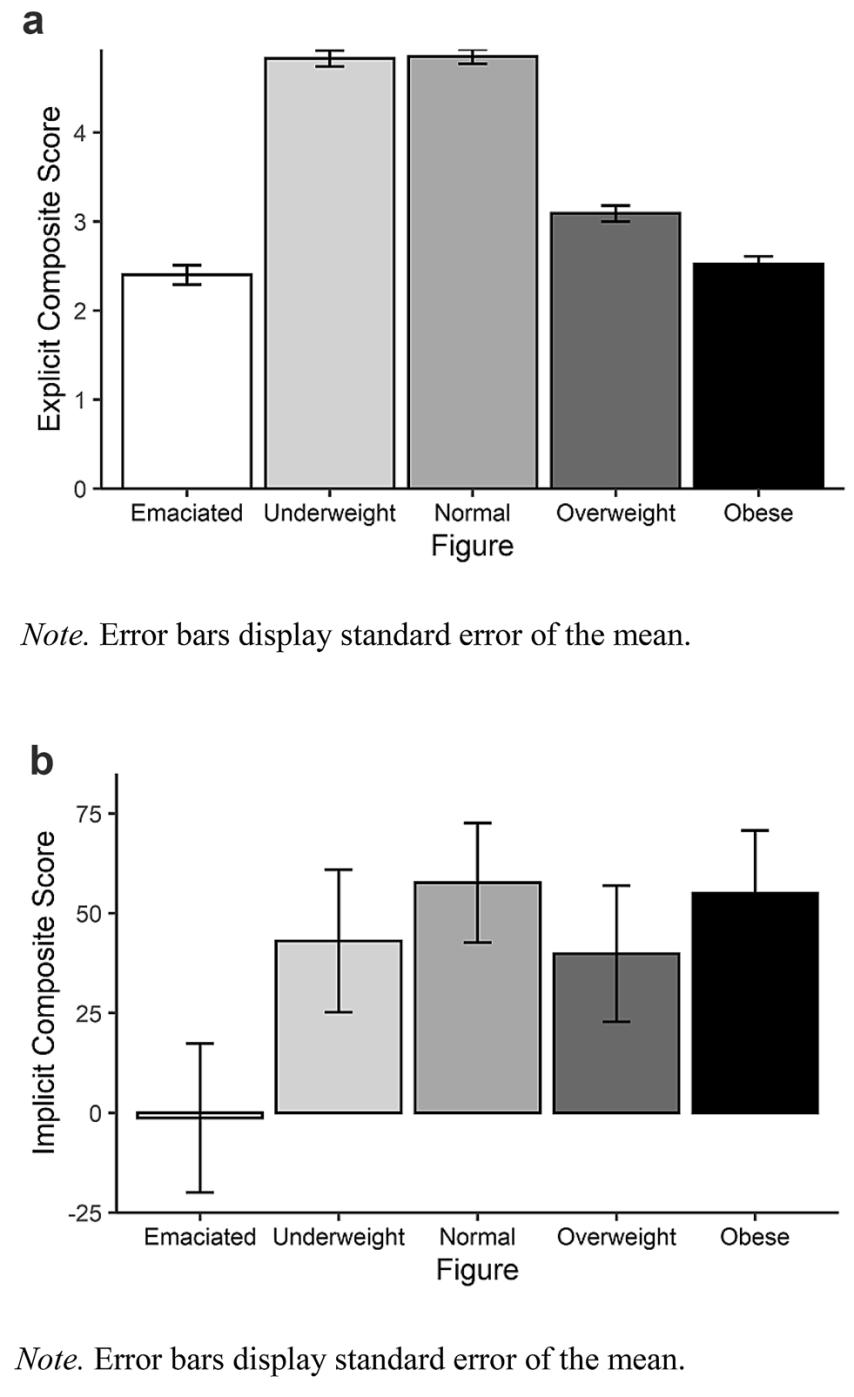
**a** Explicit evaluative responses toward the pictures of the different BMIs. **b** Implicit evaluative responses toward the pictures of the different BMIs

Regarding the composite score of the explicit attitudes, a significant main effect of “figure”, *F*(2.87, 381.80) = 256.53, *p* < 0.001, $\eta_{{\text{P}}}^{2}$ = 0.66 was found (see Fig. 3a). Repeated contrasts showed that the rating of each picture category differed from the following one (all *p* < 0.001), with one exception: there was no difference between the rating of the pictures for the underweight and normal weight women (*p* = 0.824). Furthermore, simple contrasts showed that the rating was more positive for the three middle categories compared to the rating of both extreme categories (emaciated, obese) (all *p* < 0.001). However, there was no difference between the ratings of the emaciated and obese category (*p* = 0.317). A simple t-test between the underweight and overweight category carved out a significant difference, *t*(133) = 15.66, *p* < 0.001.

Regarding the reaction time difference for the implicit evaluative response score, there was no significant main effect of “figure”, *F*(3.88, 515.82) = 1.95, *p* = 0.103, $\eta_{{\text{P}}}^{2}$ = 0.014 (see Fig. 3b).

There were no significant correlations between the explicit and implicit attitudes of the five different pictures of the BMI (all *p* > 0.05).

### Prediction of body satisfaction

Two stepwise regression analyses were computed for the two body satisfaction variables, namely the body area satisfaction scale and the appearance evaluation scale of the MBSRQ with the predictors mentioned in the method section.

The first stepwise regression showed that 31.8% (*R* = 0.564) of appearance evaluation was predicted by the BMI, the actual-ideal discrepancy, the negative scale of self-compassion and the two mindfulness aspects of acting with awareness and describing, *F*(5, 128) = 11.96, *p* < 0.001, see Table 2. Multi-collinearity was checked and none of the tolerance factors were below 0.795 and none of the variance inflation factors were above 1.259, which is tolerable (Urban & Mayerl, 2011).

**Table 2:** Regression-analysis with the criterion body appearance evaluation

Variable	*B*	95% CI for *B*		SE *B*	β	*R*^2^	Δ *R*^2^
		*LL*	*UL*				
Step 1						0.12	0.12***
Constant	3.80***	3.68	3.93	0.06			
AID	− 0.20***	− 0.29	− 0.11	0.05	− 0.35***		
Step 2						0.23	0.11***
Constant	4.64***	4.23	5.05	0.21			
AID	− 0.21***	− 0.30	− 0.13	0.04	− 0.38***		
Negative SCS	− 0.27***	− 0.40	− 0.14	0.06	− 0.33***		
Step 3						0.26	0.03*
Constant	3.90***	3.14	4.67	0.39			
AID	− 0.21***	− 0.29	− 0.13	0.04	− 0.38***		
Negative SCS	− 0.22**	− 0.35	− 0.09	0.07	− 0.26**		
AWA	0.02*	0.003	0.04	0.01	0.18*		
Step 4						0.29	0.04*
Constant	5.03***	3.89	6.17	0.58			
AID	− 0.16**	− 0.25	− 0.07	0.05	− 0.29**		
Negative SCS	− 0.23**	− 0.36	− 0.10	0.07	− 0.27**		
AWA	0.03*	0.01	0.05	0.01	0.21*		
BMI	− 0.06*	− 0.10	− 0.01	0.02	− 0.21*		
Step 5						0.32	0.03*
Constant	4.58***	3.39	5.78	0.61			
AID	− 0.15**	− 0.24	− 0.06	0.05	− 0.27**		
Negative SCS	− 0.19**	− 0.32	− 0.06	0.07	− 0.23**		
AWA	0.03*	0.01	0.04	0.01	0.20*		
BMI	− 0.06**	− 0.10	− 0.02	0.02	− 0.24**		
Describe	0.02*	0.001	0.03	0.01	0.17*		

Predictors excluded from the final model: positive scale SCS (*β* = 0.054, *p* = 0.535), implicit attitudes toward emaciated weight (*β* = 0.079, *p* = 0 .298), underweight (*β* = − 0.099, *p* = 0.183), normal weight (*β* = 0.010, *p* = 0.899), overweight (*β* = 0.034, *p* = 0.649), obese (*β* = − 0.040, *p* = 0.602), and explicit attitudes toward emaciated weight (*β* = − 0.133, *p* = 0.080), underweight (*β* = − 0.037, *p* = 0.643), normal weight (β = − 0.002, *p* = 0.982), overweight (*β* = − 0.094, *p* = 0.208), obese (*β* = − 0.055, *p* = 0.459), and social media (*β* = 0.020, *p* = 0.798), FFMQ non judging (*β* = 0.110, *p* = 0.304)

*CI* confidence interval, *LL* lower limit, *UL* upper limit, *AID* actual-ideal weight discrepancy, *Negative SCS* Negative Self-Compassion Scale, *AWA* FFMQ, acting with awareness, *BMI* body mass index, *Describe* FFMQ, describing

*p < 0.05, **p < 0.01, ***p < 0.001

Second, another multiple regression for the body area satisfaction scale showed that 16.9% (*R* = 0.412) is explained by the predictors actual-ideal weight discrepancy and the negative scale of self-compassion, *F*(2, 131) = 13.37, *p* < 0.001, see Table 3. Multi-collinearity was checked and none of the tolerance factors were below 0.992 and none of the variance inflation factors were above 1.008, which is tolerable (Urban & Mayerl, 2011).

**Table 3:** Regression-analysis with the criterion body area satisfaction

Variable	*B*	95% CI for B		SE* B*	β	*R*^2^	Δ *R*^2^
		*LL*	*UL*				
Step 1						0.10	0.10***
Constant	4.32***	3.92	4.73	0.20			
Negative SCS	− 0.25***	− 0.37	− 0.12	0.06	− 0.32***		
Step 2						0.17	0.07**
Constant	4.48***	4.08	4.88	0.20			
Negative SCS	− 0.27***	− 0.39	− 0.14	0.06	− 0.34***		
AID	− 0.14**	− 0.22	− 0.06	0.04	− 0.26**		

Predictors excluded from the final model: positive scale SCS (*β* = 0.089, *p* = 0.340), implicit attitudes toward emaciated weight (*β* = 0.125, *p* = 0 .124), underweight (*β* = − 0.100, *p* = 0.220), normal weight (*β* = 0.046, *p* = 0.563), overweight (*β* = − 0.087, *p* = 0.276), obese (*β* = − 0.084, *p* = 0.295), and explicit attitudes toward emaciated weight (*β* = − 0.076, *p* = 0.344), underweight (*β* = − 0.017, *p* = 0.833), normal weight (β = − 0.055, *p* = 0.497), overweight (*β* = − 0.113, *p* = 0.161), obese (*β* = - 0.029, *p* = 0.719), and social media (*β* = − 0.083, *p* = 0.311), BMI (*β* = − 0.079, *p* = 0.369), FFMQ acting with awareness (*β* = 0.077, *p* = 0.365), FFMQ non judging (*β* = 0.089, *p* = 0.422) and the FFMQ describe (*β* = 0.029, *p* = 0.732)

*CI* confidence interval, *LL* lower limit, *UL* upper limit, *Negative SCS* Negative Self-Compassion Scale, *AID* actual-ideal weight discrepancy

*p < 0.05, **p < 0.01, ***p < 0.001

## Discussion

In summary, our results have shown that the explicit affective attitudes toward emaciated and obese female figures are more negative than the ones toward pictures of underweight, overweight and normal weight pictures. Besides, the pictures of underweight female figures are rated as more positive than the pictures of overweight women. Regarding the implicit affective attitudes, there were no significant differences. This is partly in contrast to our first hypothesis. There were no relations between implicit and explicit measurements, which is at least in line with the second part of our first hypothesis. Regarding our second hypothesis, we only found small positive correlations between the implicit attitudes toward pictures of emaciated women and the satisfaction with different body areas. Concerning our third hypothesis, the predicting factors for the body appearance evaluation scale were the BMI, the actual-ideal weight discrepancy, the negative scale of self-compassion and the mindfulness aspects acting with awareness and describing. For the body area satisfaction scale, the actual-ideal weight discrepancy and the negative scale of self-compassion were the significant predictors.

### Implicit and explicit affective attitudes toward under- and overweight

In contrast to the results of Roddy et al. (2009), who found a positive implicit pro-slim effect, but partly in line with the study of Maroto Expósito et al. (2015), an explicit positive pro-slim bias was found when taking into account the rating toward under- and overweight and not the extreme versions (emaciated, obese) of these categories. The authors did not differentiate between different forms of under- and overweight, they were only using pictures from women with a BMI below 18.5 and above 25 (Maroto Expósito et al., 2015). Consequently, our results extend their findings, showing that the explicit pro-slim bias only exists when the low BMI was still seen as healthy. If a picture of an underweight woman with a pathologically low BMI was shown, the explicit attitude toward the woman was as low as the explicit affective attitude toward a picture of an obese women. The results of the implicit affective priming task might differ from the study of Roddy et al. (2009) due to the different implicit measurements used. The authors used the IAT, as well as the IRAP and considered pictures of women and men who were either overweight or normal weight (Roddy et al., 2009). They did not investigate the affective implicit attitudes and did also not differentiate between different BMI categories. Also, in contrast to our study, men participated. Next to the methodological differences between both studies one might speculate that the more cognitive implicit measurement could be more related to body satisfaction, because body satisfaction has a higher cognitive instead of emotional component (Arbour & Ginis, 2006). The lack of correlations between the explicit and implicit measurements is not surprising, because in a meta-analyses also only a relation of 0.13 could be detected between implicit and explicit attitudes (Cameron et al., 2012). Applying the affective misattribution procedure might have resulted in a higher relation between explicit and implicit measurements (Cameron et al., 2012). The missing relation between implicit and explicit affective attitudes and body satisfaction contradicts on the one hand with the study of Hernández-López et al. (2019) but is on the other hand in line with the study of Watts et al. (2008). The authors of this study did not find a relation between individual difference variables, like an appearance rating and an affective priming task, as well. The different results in the study of Hernández-López et al. (2019) could again be attributed to the different methodology, first due to the application of the IRAP and second due to the investigation of women with extreme forms of body dissatisfaction only. Consequently, it is necessary to take the used implicit measurement into account when discussing about implicit attitudes toward body images. Also, the different forms of under- and overweight should be considered. And furthermore, the method of assessing body satisfaction should be considered. In this study, we used for example the body satisfaction area scale where a cognitive judgment but not an affective evaluation of the own satisfaction of some body parts was retrieved, as already mentioned above.

### Factors which are related to body satisfaction

The detection of the predicting mindfulness variables (awareness and describing) is in line with the study of Prowse et al. (2013). Even though the authors used another mindfulness questionnaire (Kentucky Inventory of Mindfulness Skills) as well as another Body Image Satisfaction Questionnaire (Body Image Acceptance and Action Questionnaire), the results are comparable. In both studies the mindfulness aspect of “observing” is not relevant for a better body image resulting in the assumption that an enhanced ability of observing might also lead the attention to body parts, which are not perceived as perfect. Furthermore, the predicting role of the negative scale of self-compassion for the body appearance evaluation scale and the body-area satisfaction scale is in line with a review article concerning the relation between eating disorders and self-compassion (Braun et al., 2016). It is a new finding that only the negative scale of self-compassion is relevant for detecting a relation to some aspects of body image. However, in a study with German football players, it has also been shown that only the negative but not the positive scale of self-compassion was related to psychological variables (Jansen, 2021), which gives a hint, that the relevance of both scales has to be investigated in more depth. Finally, the predicting factor of the actual-ideal weight discrepancy on body image is in line with the study of Swami et al. (2008). In the study presented here, we could show that this predicting factor is relevant for both MBSRQ scales chosen here, the body appearance evaluation scale, as well as the body area satisfaction scale. As it is stated by the authors of the German version, the subscales of the MBSRQ are highly interrelated, but it seemed to be reasonable to have a measurement to distinguish between the feelings toward the own whole body and the ratings of single body parts (Vossbeck-Elsebusch et al., 2014). Smeets et al. (1997) mention perceptional distortion measurements, which found larger effect sizes with whole-body techniques compared to body-parts techniques.

Furthermore, our results did not show strong relations of social media use and body satisfaction as suggested by a systematic review (Holland & Tiggemann, 2016). The results of our study are also contrary to a study with undergraduates, where it was shown that the length of time spent on Facebook per day, the number of Facebook friends, as well as the emotional investment in Facebook was related to appearance evaluation and appearance orientation (Rutledge et al., 2013). The emotional investment in Facebook was related to the appearance orientation and more Facebook friends were related to a more positive view of appearance. The only significant positive correlation of appearance orientation in this study was with “Facebook attitudes” (emotional investment in Facebook). Appearance orientation describes the overall investment in appearance in contrast to the overall satisfaction of appearance (appearance evaluation) (Vossbeck-Elsebusch et al., 2014). Both factors measure different concepts. The minor relation between social network use and body image might be due to a different basis rate of using social networks, because it has been shown that network sizes differ significantly between people from the US and Germany (Brake, 2017).

### Limitations

First of all, the study presented here was implemented as an online study and no correlational study has been done to a lab-based approach. However, in cognitive psychology, several classical effects have been successfully replicated in online experiments (Kochari, 2019), leading to the assumption that the online measurement used here is acceptable. For the investigation of the implicit affective attitudes, an affective priming-paradigm has been chosen which is only one possible implicit measurement out of many. The relation to the former used implicit measurements in this area (IAT and IRAP) should be investigated in detail to make the results more comparable to previous studies. Unfortunately, the pictures from the PFRS were only female, so only women could participate. This limits the generalizability of the results. Also, the order of the questionnaires might have influenced the explicit and implicit attitudes of the women participating. Finally, due to the correlational nature of the study, no causal conclusions could be drawn.

## Conclusion

The strength of the study was the investigation of the relation of two different aspects of affective attitudes toward different body shapes. This study confirms an explicit positive affective bias toward pictures of slim persons and a negative bias toward emaciated and obese body pictures. The new aspect is that on an explicit level the extreme categories were rated as less positive, but on an implicit level there were no differences between the categories. Accordingly, one future research question might be to investigate why there were no implicit biases toward extreme categories. How relevant are expectations to give a socially appropriate answer while assessing explicit attitudes? Furthermore, implicit and explicit affective attitudes toward different body shape categories were not interrelated and most importantly for this study, affective attitudes were not related to body satisfaction. However, our study confirms the role of different aspects of mindfulness and self-compassion, as well as the actual-ideal weight discrepancy and the own BMI for body satisfaction.
